# Supplementary material for: A Survey of 1000 Respondents on the Polish Population’s Knowledge and Attitudes about Tissue/Organ Donation and Transplantation in Times of Allogeneic Tissue Shortage
Source: Int J Environ Res Public Health. 2022 Oct 25;19(21):13875. doi: 10.3390/ijerph192113875 (PMC9654482; doi:10.3390/ijerph192113875)
Supplement: Supplementary file 1 [file ijerph-19-13875-s001.zip › ijerph-1894475-supplementary.pdf]

Organ and tissue transplant survey

Good morning! We present you a questionnaire on knowledge and attitudes towards organ and tissue transplantation in Poland. We ask for diligent answers to the questions included in the survey. It is anonymous. It consists of 6 questions about the metric and 17 essential questions. Thank you.

1. Please select your age:

Select only one answer.

16 - 20 years

21 - 30 years

31 - 40 years old

41 - 50 years

51 - 60 years

61 - 70 years

over 70 years old

2. Please select your education:

Select only one answer.

Basic

Vocational

Medium

Higher

3. Please select the place of residence:

Select only one answer.

Village

A city with up to 50,000 inhabitants

A city with up to 100,000 inhabitants

A city with up to 200,000 inhabitants

A city with over 200,000 inhabitants

4. Please select the type of education:

Select only one answer.

Medical

Not related to medicine

5. Please select your gender:

Select only one answer.

Man

Woman

6. Have you been / have you been a patient of the dr S. Sakiel Burn Treatment Center for them. in Siemianowice Śląskie?

Select only one answer.

Yes

No

QUESTIONAIRE

1. Do you think that retrieval of an organ from a deceased person can save the life of another living person?

   Select only one answer.

   Yes

   No

   I do not know

2. Has any of your relatives become a tissue or organ donor after their death?

   Select only one answer.

   Yes

   No

   I do not know

3. Would you agree to donate your organs after DEATH?

   Select only one answer.

   Yes

   No

   I do not know

4. Would you agree to become a donor of your organs during LIFE?

   Select only one answer.

   Yes

No

I do not know

5. Would you consent to the transplantation of an organ / tissue / cells derived from a deceased person in a situation of threat to your life or health?

   Select only one answer.

   Yes

   No

   I do not know

6. To become a donor after your death, according to Polish law, you should:

   Select only one answer.

   Not to object in life to organ donation (implicit consent)

   Give your consent in writing to the removal of tissues and organs, e.g. through the so-called A "declaration of will" or a Transplant card with the Gift of Life.

   Register as a potential donor with the Central Donor Register

7. Do you think that a person that refuses to consent to the retrieval of tissues and organs after their death should receive a transplant in a situation where their life or health is at risk?

   Select only one answer.

   Yes

   No

   I do not know

8. Tissues and organs are retrieved:

   Select only one answer.

   after finding permanent irreversible cessation of brain activity by a team appointed especially for this circumstance

   when the cardiorespiratory action has stopped for more than 45 minutes

   after an unsuccessful resuscitation action lasting min. 60 minutes

   after confirming brain death based on the results of the double EEG test

9. What organs would you consent to to donate?

   Select all correct answers.

   heart

   the kidneys

   liver

   lungs

   none

   all

10. The statements of permanent irreversible cessation of brain activity are made by:

    Select only one answer.

    specialists in the fields of medicine: anesthesiology and intensive care, neurology,

    neurosurgery and forensics,

    court with jurisdiction over the potential donor's place of residence based on expert opinions

    attending physician based on the physical examination and medical history

    transplant coordinator

11. The collection of tissues and organs from a deceased donor takes place in:

Select only one answer.

the hospital in which it was found that permanent and irreversible cessation of brain activity was found

in the dissecting room

at the funeral home

in a tissue bank

12. What tissues would you consent to donating?

Select all correct answers.

cornea (eye)

skin

bones

tendons

none

all

13. The role of the family in the process of selecting a potential donor consists in:

Select only one answer.

consents to the removal of tissues and organs from the deceased person

confirms the lack of objection expressed during the life of the deceased person

the family does not play any role in the process of selecting a potential donor

only a family member authorized in the medical history may make a decision after the death of a potential donor

14. How does religion influence the decision to donate organs and tissues after death?

Select only one answer.

my religion categorically forbids the donation of tissues and organs after death

my religion allows for the donation of tissues and organs after death

my religion has no bearing on my decisions regarding the donation of tissues and organs after death

I am a non-believer and I do not consider religion as a decisive factor in the donation of tissues and organs after death

15. Would you consent to skin removal from your deceased relative?

Select only one answer.

no, because I believe it is a desecration and mutilation of a corpse

yes, because skin grafts can save lives

I would have followed the deceased's will

the decision would be made after agreement with the family

16. Cosmetic activities performed by collection teams, after the collection of tissues and organs, consist of:

Select only one answer.

ensuring the natural, anatomical appearance of the cadaver - just like before the collection

collection teams do not perform any cosmetic activities - the body is transferred to a funeral home

the body must be cremated after the tissues and organs are removed

17. Have you ever talked to your relatives about the collection of tissues and organs?

Select only one answer.

Yes

No

I do not remember
